# Production of Anti-LPS IgM by B1a B Cells Depends on IL-1β and Is Protective against Lung Infection with *Francisella tularensis* LVS

**DOI:** 10.1371/journal.ppat.1004706

**Published:** 2015-03-13

**Authors:** Laura del Barrio, Manoranjan Sahoo, Louis Lantier, Joseph M. Reynolds, Ivonne Ceballos-Olvera, Fabio Re

**Affiliations:** Department of Microbiology & Immunology, Chicago Medical School, Rosalind Franklin University of Medicine and Science, North Chicago, Illinois, United States of America; Stanford University School of Medicine, UNITED STATES

## Abstract

The role of IL-1β and IL-18 during lung infection with the gram-negative bacterium *Francisella tularensis* LVS has not been characterized in detail. Here, using a mouse model of pneumonic tularemia, we show that both cytokines are protective, but through different mechanisms. *Il-18^-/-^* mice quickly succumb to the infection and showed higher bacterial burden in organs and lower level of IFNγ in BALF and serum compared to wild type C57BL/6J mice. Administration of IFNγ rescued the survival of *Il-18^-/-^* mice, suggesting that their decreased resistance to tularemia is due to inability to produce IFNγ. In contrast, mice lacking IL-1 receptor or IL-1β, but not IL-1α, appeared to control the infection in its early stages, but eventually succumbed. IFNγ administration had no effect on *Il-1r1^-/-^* mice survival. Rather, *Il-1r1^-/-^* mice were found to have significantly reduced titer of *Ft* LPS-specific IgM. The anti-*Ft* LPS IgM was generated in a IL-1β-, TLR2-, and ASC-dependent fashion, promoted bacteria agglutination and phagocytosis, and was protective in passive immunization experiments. B1a B cells produced the anti-*Ft* LPS IgM and these cells were significantly decreased in the spleen and peritoneal cavity of infected *Il-1b^-/-^* mice, compared to C57BL/6J mice. Collectively, our results show that IL-1β and IL-18 activate non-redundant protective responses against tularemia and identify an essential role for IL-1β in the rapid generation of pathogen-specific IgM by B1a B cells.

## Author Summary

*Francisella tularensis* is a Gram-negative bacterium that infects macrophages and other cell types causing tularemia. *F*. *tularensis* is considered a potential bioterrorism agent and is a prime model intracellular bacterium to study the interaction of pathogens with the host immune system. The role of the proinflammatory cytokines IL-1β and IL-18 during lung infection with *F*. *tularensis* has not been characterized in detail. Here, using a mouse model of pneumonic tularemia, we show that both cytokines are protective, but through different mechanisms. Mice deficient in IL-18 quickly succumbed to the infection but administration of IFNγ rescued their survival. In contrast, mice lacking IL-1β appeared to control the infection in its early stages, but eventually succumbed and were not rescued by administration of IFNγ. Rather, IL-1β-deficient mice had significantly reduced serum level of IgM antibodies specific for *F*. *tularensis* LPS. These antibodies were generated in a IL-1β-, TLR2-, and ASC-dependent fashion, promoted bacteria agglutination and phagocytosis, and were protective in passive immunization experiments. B1a B cells produced the anti-*F*. *tularensis* IgM and were significantly decreased in the spleen and peritoneal cavity of infected IL-1β-deficient mice. Collectively, our results show that IL-1β and IL-18 activate non-redundant protective responses against tularemia and identify an essential role for IL-1β in the rapid generation of pathogen-specific IgM by B1a B cells.

## Introduction

*Francisella tularensis* (*Ft*) is a Gram-negative bacterium that infects macrophages and other cell types causing tularemia [1]. *Ft* is considered a potential bioterrorism agent and is used as a prime model intracellular bacterium to study the strategies adopted by microbes to evade and minimize innate immune detection.

Although the innate immune response to *Ft* infection has been examined in a great number of publications (reviewed in [2] [3]), much remains to be learned. *Ft* is known to evade various host defense mechanisms [4] and to produce an atypical LPS that does not stimulate TLR4 and does not possess proinflammatory activity [5] [6] [7,8] [9]. However, like others, we have shown that *Ft* stimulates a proinflammatory response primarily through TLR2 [10] [11] [12], which recognizes *Ft* lipoproteins [13]. The other innate immune pathway preferentially stimulated by *Ft* in mice is the inflammasome composed of AIM2-ASC-caspase-1 [14]. It is believed that genomic DNA released by lysing bacteria localized in the cytosol activates this inflammasome, leading to secretion of IL-1β and IL-18 and death of the infected cells by pyroptosis. This form of caspase-1-dependent cell death has been shown to effectively restrict intracellular replication of several bacteria, including *Ft*, by exposing them to extracellular microbicidal mechanisms [15]. Activation of the NLRP3 inflammasome in human macrophages has also been reported [16]. Whether this inflammasome is also activated in mice is unclear.

IL-1β and IL-18 are powerful proinflammatory cytokines that have been shown to be protective in a large number of experimental infection models. Both cytokines signal through the MyD88 pathway but elicit varied responses in different cell types [17]. Despite intensive research on *Ft*, the role of IL-1β and IL-18 during lung infection with this bacterium has not been characterized in detail. A confounding factor that affects the tularemia research field is that the majority of the studies are performed using either of two *Francisella* subspecies, *F*. *novicida* or *Ft* live vaccine strain (LVS), which are pathogenic in mice, but not humans, and differentially engage innate immune responses [1] [18]. An additional strain, the virulent *Ft* type A SchuS4 strain, displays an exaggerated virulence in mice, which has severely limited its use for the genetic analysis of the host immune response to this infection. A further complication in the analysis, comparison, and interpretation of the studies on tularemia, is that different routes of infection (i.p., i.d., i.n.) are used, which determine the severity of disease, and the relative contribution to protection of various immune pathways [19] [20]. For our studies we decided to use LVS because of its potential use as prophylactic vaccine and we adopted the intranasal infection route because it is the most lethal and the most relevant to biodefense.

Early evidence that IL-1β and IL-18 played protective roles during infection with *Francisella* species was provided by studies of Denise Monack’s group that showed that intraperitoneal injection of neutralizing antibodies against IL-18 and IL-1β increased bacterial burdens in mice infected intradermaly with *F*. *novicida* [21]. The same group showed that mice deficient in both IL-1β /IL-18 are more susceptible than C57BL/6J mice, yet not as much as mice deficient in ASC or caspase-1, suggesting that other caspase-1-dependent pathways, most likely pyroptosis, significantly contributed to protection from infection [14]. Collazo *et al*. analyzed *Il-1r1* ^-/-^ and *Il-18* ^-/-^ mice infected intradermally with *Ft* LVS, but did not find significant differences in the survival of these mice, compared to C57BL/6J mice [22]. Collectively, these studies indicated that IL-1β and IL-18 may contribute to protection from tularemia but it remains unclear to what extent, through which mechanism, and under which conditions. Despite the uncertainties about the role of IL-1β and IL-18 during *Ft* infection, several groups, including ours, have shown that *Ft* mutants that hyper-activate the inflammasome leading to increased IL-1β and IL-18 secretion are attenuated in vivo [23] [24] [25] [26,27], suggesting that these cytokines in fact play a protective role in tularemia.

The studies described here analyzed for the first time the response of IL-1- or IL-18-deficient mice to intranasal infection with *Ft* LVS. Our results demonstrate that different protective mechanisms are activated by IL-18 and IL-1β and reveal a critical role for IL-1β in the production of anti-*Ft* LPS IgM by B1a B cells.

## Results

### Different susceptibility of *Il-1r1* ^-/-^ and *Il-18* ^-/-^ mice to *Ft* LVS lung infection

**Fig 1:**
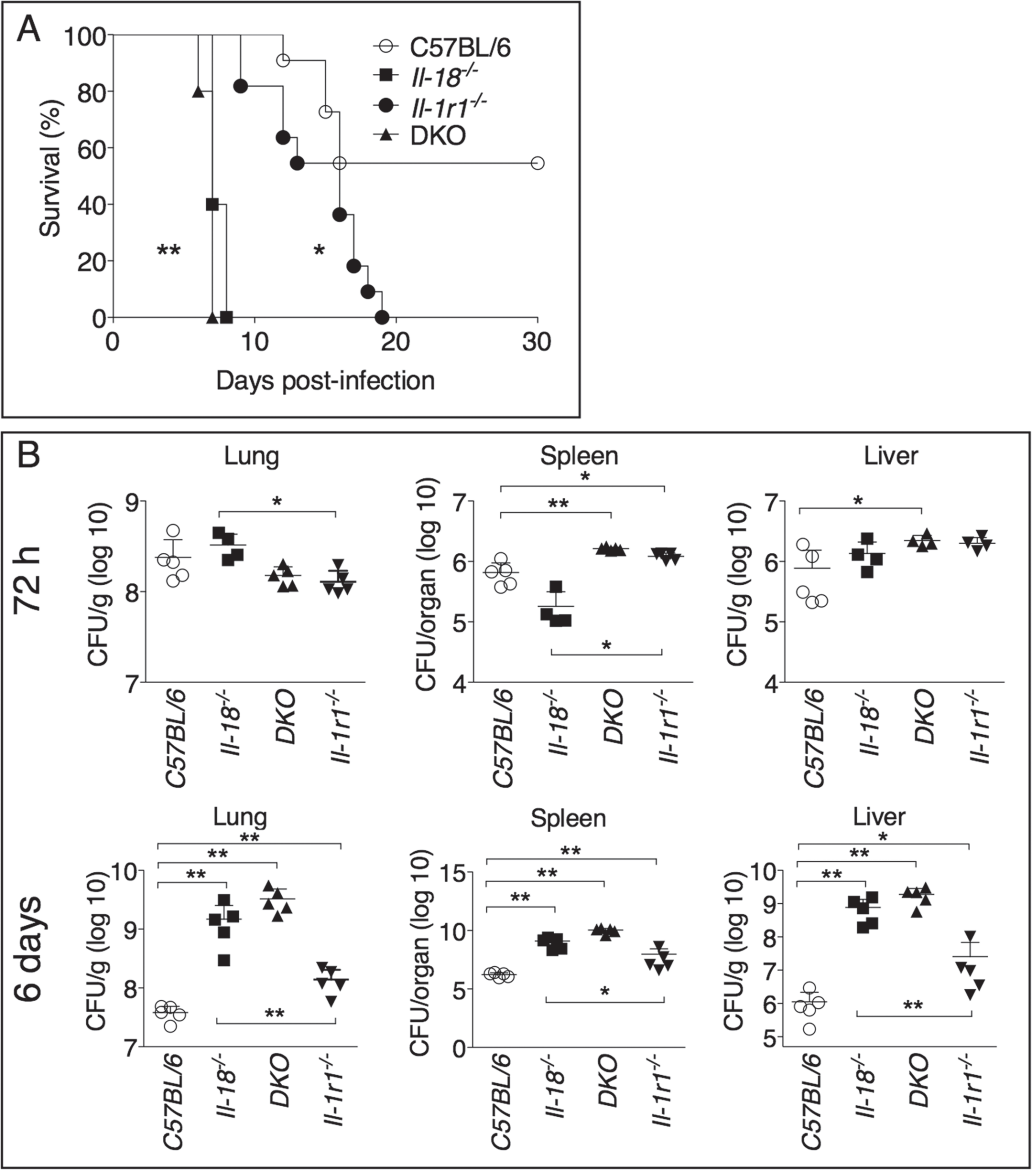
IL-1RI and IL-18-deficient mice are more susceptible to lung infection with *Ft* LVS. (A) C57BL/6J or *Il-1r1* ^-/-^ mice (*n =* 11), *or Il-18* ^-/-^ and *Il-1r1* ^-/-^/*Il-18* ^-/-^ (DKO) mice (*n* = 5) were intranasally infected with 4x10^3^ CFU *Ft* LVS and survival was monitored. (B) Mice infected as in A were euthanized 72 hours or 6 days p.i. and bacterial burden in organs was measured. One representative experiment of two is shown. Data are expressed as mean + S.D. *p<0.05, **p<0.01. (B) Mann-Whitney U test.

To increase our understanding of the role of IL-1 and IL-18 during lung infection with *Ft* LVS, we intranasally infected mice deficient in the IL-1 receptor, *Il-1r1* ^-/-^, or mice deficient in IL-18 and measured their survival. As shown in Fig. 1A, both mouse strains were significantly more susceptible than the wild type C57BL/6J mice. Mice deficient in both IL-1 receptor and IL-18 (DKO) were also more susceptible. Interestingly, the mean time to death of *Il-18* ^-/-^ mice was much shorter than that of *Il-1r1* ^-/-^ mice, an observation that may suggest either that IL-18 plays a more critical role than IL-1 in this model of infection, or that each cytokine may be required at a different time point during infection (see below). While the bacterial burden in organs 72 hours post infection were not consistently increased in the knock-out mouse strains compared to C57BL/6J mice, 6 days p.i. *Il-18* ^-/-^ mice had significantly higher burdens than *Il-1r1* ^-/-^ mice (Fig. 1B) confirming the relative contribution of each cytokine. Compared to other gram-negative bacterial infection models, infection and innate immune response occur with relatively slower kinetics during tularemia and this may explain why the differences in bacterial burdens become more evident at later time points.

### Role of IL-18 during lung infection with *Ft* LVS

**Fig 2:**
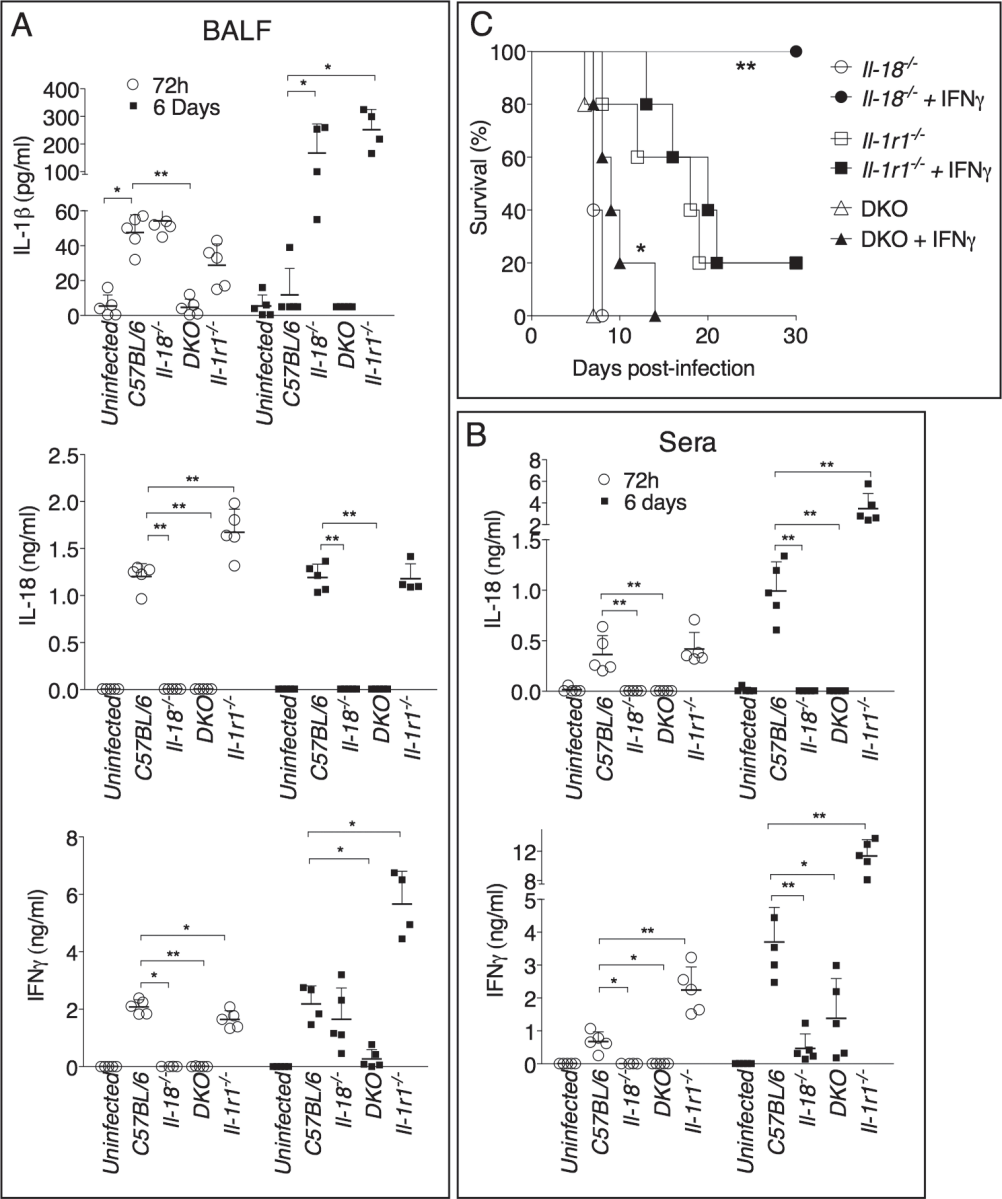
IFNγ administration rescues survival of *Il-18* ^-/-^, but not *Il-1r1* ^-/-^, mice intranasally infected with *Ft* LVS. (A) Cytokine levels were measured 72 hours or 6 days p.i. in BALF (A) or serum (B) of mice intranasally infected with 4x10^3^ CFU *Ft* LVS. (C) Mice (*n* = 5), of shown genotypes, were intranasally infected with 4x10^3^ CFU *Ft* LVS and recombinant IFNγ (1 μg) was administered daily by i.p. injection for the first 6 days. Survival was monitored. One representative experiment of two is shown. Data are expressed as mean + S.D. *p<0.05, **p<0.01. Mann-Whitney U test.

IL-1β and IL-18 levels were measured in the bronchoalveolar lavage fluid (BALF) or serum obtained from infected mice 72 hours or 6 days post-infection (Fig. 2A, B). IL-1β was not detectable in the sera of infected mice, but it was detected in higher amount at 72 hours than at 6 days p.i. in the BALF of C57BL/6J mice, a pattern that paralleled the reduction in bacterial burdens (see Fig. 1B). Production of IL-1β was drastically increased in *Il-1r1* ^-/-^ and *Il-18* ^-/-^ mice. Both IL-1 and IL-18 are known to induce *Il-1b* mRNA [28], which may explain why IL-1β level in BALF of DKO mice was severely reduced, compared to the other strains, despite high bacterial burden. IL-18 was detected in higher amounts in BALF, at 72 h, or in sera, at 6 days, in *Il-1r1* ^-/-^ mice, compared to C57BL/6J mice. The levels IFNγ, a cytokine that is known to be protective during several bacterial infections, including tularemia [29] [30] [31] [32] [33], were reduced in *Il-18* ^-/-^ mice, a finding consistent with the established function of IL-18 as an IFNγ-inducing cytokine [29], but were significantly increased in BALF or sera of *Il-1r1* ^-/-^ mice, a likely reflection of the high level of IL-18 in these mice. Collectively, these results suggest that the reduced resistance of *Il-18* ^-/-^ mice may be due to inability to produce sufficient IFNγ. This hypothesis was confirmed by the observation that administration of recombinant IFNβ to *Il-18* ^-/-^ mice during the first 6 days of infection completely rescued their survival (Fig. 2C). In contrast, IFNγ administration did not affect the survival of *Il-1r1* ^-/-^ mice, whose BALF already contained sustained level of IFNγ. These results show that distinct protective mechanisms are triggered by IL-18 and IL-1β during lung infection with *Ft* LVS. Interestingly, IFNγ administration to DKO mice significantly improved their survival but not to the extent of *Il-1r1* ^-/-^ mice, suggesting that IL-18 may have other protective functions not strictly related to induction of IFNγ.

### IL-1β, not IL-1α, mediates protection during *Ft* LVS infection and is important in the early phase of the infection

**Fig 3:**
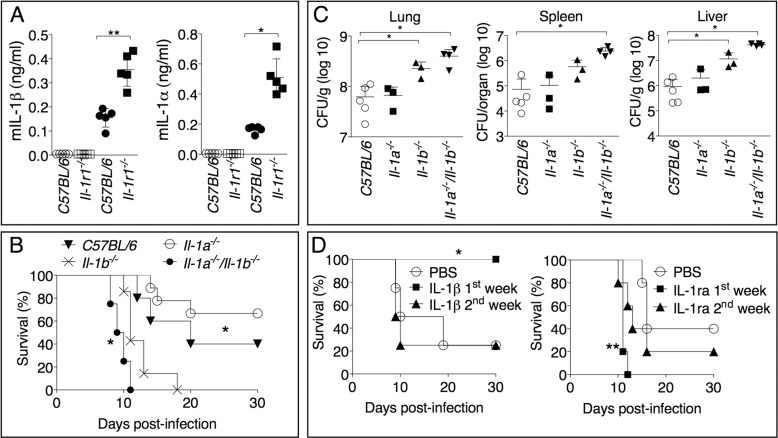
IL-1β, not IL-1α, is protective and acts in the early phase of *Ft* LVS intranasal infection. (A) IL-1α or IL-1β levels were measured 72 hours p.i. in BALF of mice intranasally infected with 4x10^3^ CFU *Ft* LVS (solid symbols) or mock infected (empty symbols). (B) Mice (*n* = 5), of shown genotypes, were intranasally infected with 4x10^3^ CFU *Ft* LVS and survival was monitored. * C57BL/6J or *Il-1a* ^-/-^/*Il-1b* ^-/-^ compared to *Il-1b* ^-/-^. (C) Organ bacterial burdens were measured 6 days p.i. in the indicated mouse strains infected as in B. (D) *Il-1b* ^-/-^ mice intranasally infected with 4x10^3^ CFU *Ft* LVS received daily intraperitoneal injections of recombinant IL-1β (1 μg). In a similar fashion, C57BL/6J mice were treated with IL-1ra (4 mg) and infected. One representative experiment of two is shown. Data are expressed as mean + S.D. *p<0.05, **p<0.01, ***p<0.001. Mann-Whitney U test.

The IL-1RI mediates response to both IL-1α and IL-1β. Although both cytokines are produced during infection with *Ft* LVS (Fig. 3A), *Il-1b* ^-/-^, but not *Il-1a* ^-/-^, mice were found to be more susceptible than C57BL/6J mice to *Ft* LVS infection (Fig. 3B). In agreement with this result, bacteria burdens were significantly increased in organs of *Il-1b* ^-/-^, but not *Il-1a* ^-/-^, mice, compared to C57BL/6J mice (Fig. 3C), confirming the protective role of IL-1β. Interestingly, the combined absence of IL-1α and IL-1β appeared to result in a more drastic phenotype than the sole absence of IL-1β. Although the bacterial burden in organs was not significantly different between *Il-1a* ^-/-^/*Il-1b* ^-/-^ and *Il-1b* ^-/-^ mice, absence of both cytokines significantly decreased mice survival compared to *Il-1b* ^-/-^ mice suggesting that, in absence of IL-1β, IL-1α may have a protective role. This effect is likely due to the fact that both cytokines are known to engage in compensatory mechanisms [34] [35] so that, in absence of IL-1β, the contribution of IL-1α, that is negligible in wild type mice, become somewhat more relevant.

As shown in Fig. 2A, production of IL-1β in C57BL/6J mice is higher at 72 h than at 6 days p.i., even though the morbidity and mortality caused by its absence becomes apparent several days later. To test the hypothesis that IL-1β is protective when produced in the early phase of the infection but not in the late phase, recombinant IL-1β was administered daily during the first week or the second week post infection to *Il-1r1* ^-/-^ mice. As shown in Fig. 3D, exogenous IL-1β was found to be protective only when administered during the first six days post infection. Conversely, blockage of IL-1, through administration of the IL-1 receptor antagonist IL-1ra to C57BL/6J mice, was deleterious during the first week but not during the second week post infection.

### IL-1β is required for production of protective IgM

Our results so far indicate that IL-1β is produced and exerts its protective effect in the early phase of the infection, but morbidity and mortality starts to become apparent in infected *Il-1r1* ^-/-^ mice only in the second week post-infection when mice definitively loose the ability to contain the infection and bacteria burden dramatically increase. This is in clear contrast to *Il-18* ^-/-^ mice that shows signs of morbidity and mortality in the early days of infection (Fig. 1A). These results, together with the observation that IFNγ administration failed to rescue *Il-1r1* ^-/-^ mice (Fig. 2C), suggest that the innate immune response of *Il-1r1* ^-/-^ mice is sufficient to effectively control the infection and protect the mice for several days. Rather, the eventual death of *Il-1r1* ^-/-^ mice may be due to failure of immune responses that are dependent on the presence of IL-1β in the early phase of infection but are effectively deployed only in the second week of infection. These features are consistent with immune responses that bridge innate and adaptive immunity, such as the generation of IgM.

**Fig 4:**
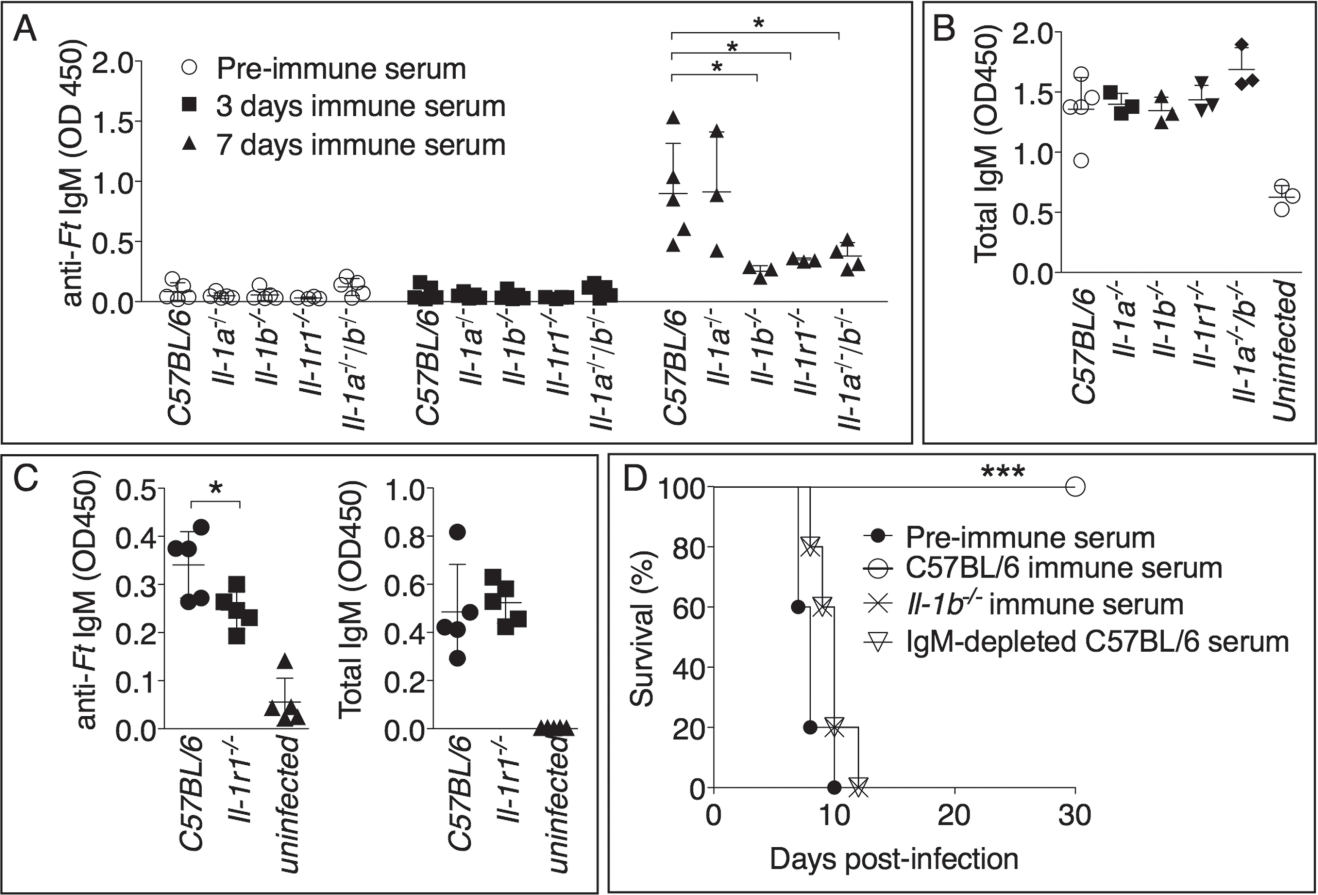
Rapid generation of protective anti-*Ft* IgM is dependent on IL-1β. Mice, of shown genotypes, were intranasally infected with *Ft* LVS 10^3^ CFU and bled at 0, 3, or 7 days p.i. *Ft*-specific (A) or total IgM titers (B) were measured in serum or BALF (C). (D) C57BL/6J mice (*n* = 5) were injected intraperitoneally with 300 μl of preimmune serum, or the indicated immune sera. 24 hours later, mice were intranasally infected with 8x10^3^ CFU *Ft* LVS and survival was monitored. One representative experiment of three (A, B) or two (C, D) is shown. Data are expressed as mean + S.D. **p<0.01, ***p<0.001. Mann-Whitney U test.

IgM is among the earliest immune effector mechanisms produced during infection and plays an important role during bacterial infections [36], including tularemia. As shown in Fig. 4A, *Ft*-specific IgM started to appear in the serum of infected mice 7 days post infection. Remarkably, the level of *Ft*-specific IgM was significantly reduced in *Il-1b* ^-/-^, *Il-1b* ^-/-^-*Il-1a* ^-/-^, or *Il-1r1* ^-/-^ mice compared to C57BL/6J or *Il-1a* ^-/-^ mice, suggesting that the susceptibility of IL-1-deficient mice may be due to insufficient production of pathogen-specific IgM and demonstrating for the first time that IL-1β is required for optimal production of this antibody isotype. The total serum IgM levels were similar among the different mouse strains (Fig. 4B) whereas the titers of *Ft*-specific IgG in serum or IgA in BALF of infected mice were undistinguishable from those of non-infected mice. (S1 Fig.). Importantly, anti-Ft IgM was also present in the BALF of infected mice (Fig. 4C). Supporting the hypothesis that reduced titer of *Ft*-specific IgM is responsible for the susceptibility of *Il-1r1* ^-/-^ mice, passive transfer of serum obtained from *Ft LVS* infected C57BL/6J mice seven days p.i. protected naïve C57BL/6J mice from infection with lethal doses of *Ft* LVS (Fig. 4D). Transfer of pre-immune serum did not confer protection, suggesting that innate antibodies were not mediating the observed protection. Remarkably, the serum obtained from *Il-1b* ^-/-^ mice was not protective, supporting the hypothesis that the observed protection was mainly mediated by *Ft*-specific IgM generated in an IL-1-dependent way. This was confirmed by the observation that serum of infected C57BL/6J mice depleted of IgM lost its protective activity. This result ruled out the possibility that other antimicrobial molecules produced during infection could play a role in the observed protection. Moreover, before passive transfer, sera were dialyzed using 100 kDa cut-off membranes to eliminate cytokines and other small inflammatory mediators.

### Anti-*Ft* IgM generation is dependent on TLR2 and ASC

**Fig 5:**
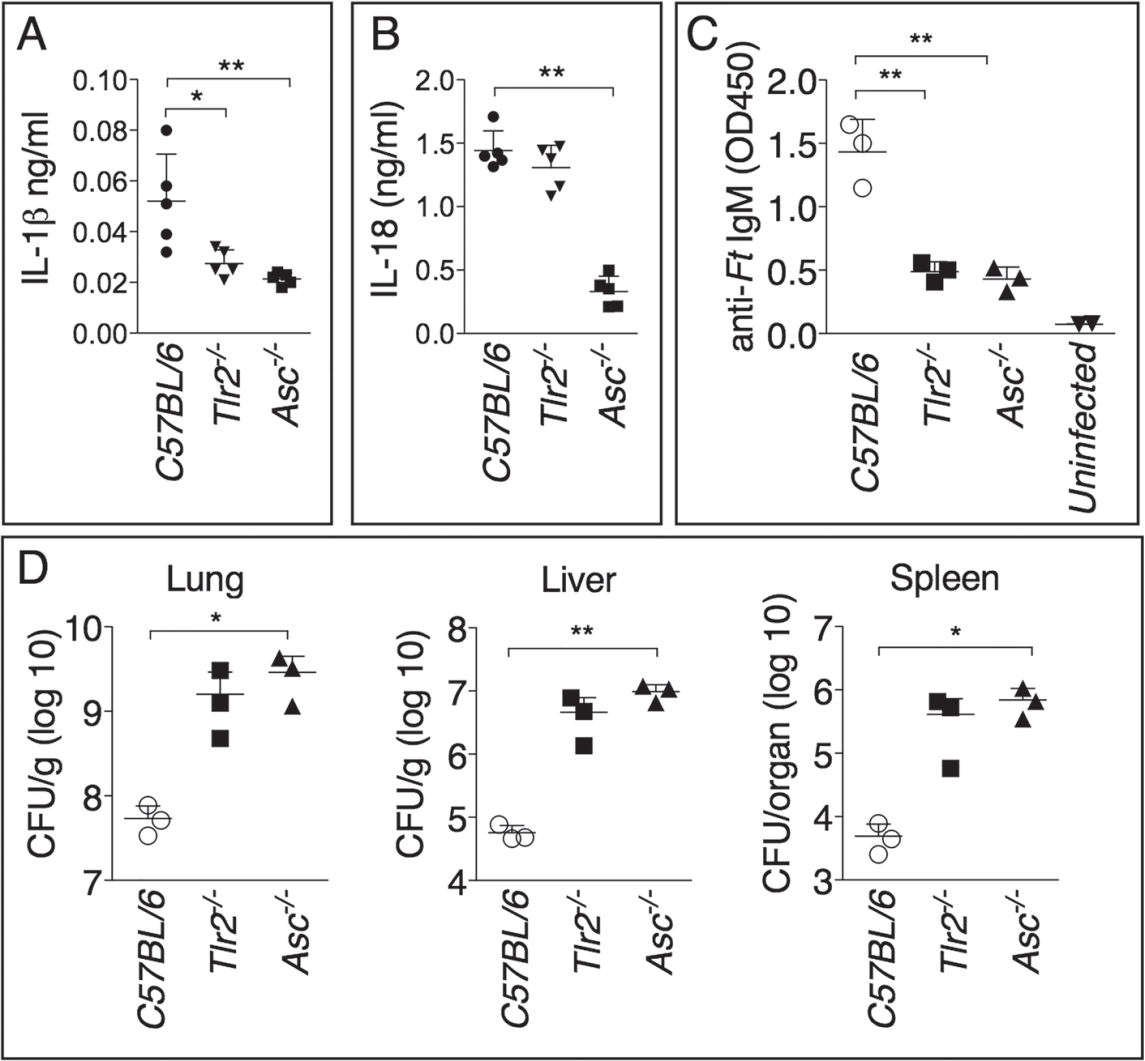
Generation of protective anti-*Ft* LPS IgM is dependent on TLR2 and inflammasome. Cytokine levels were measured 6 days p.i. in BALF (A) or serum (B) of mice intranasally infected with 4x10^3^ CFU *Ft* LVS. (C) Anti-*Ft* IgM were measured on day 7 p.i. in serum of mice infected as in A. (D) Organ bacterial burdens were measured 6 days p.i. in the indicated mouse strains infected as in A. Data are expressed as mean + S.D. *p<0.05, **p<0.01. Unpaired t-test.

As shown in Fig. 5A, and in agreement with previous works [12,21], production of mature IL-1β in response to *Ft* LVS infection is dependent on TLR2 and the inflammasome adaptor ASC. Interestingly, production of IL-18 was dependent on ASC but not TLR2 (Fig. 5B). Differently from IL-1β, IL-18 is known to be constitutively expressed, which may relieve the necessity of TLR-mediated priming steps. Reinforcing the notion that IL-1β is required for the production of anti-*Ft* IgM, the level of this antibody was significantly decreased in the serum of infected *Tlr2* ^-/-^ and *Asc* ^-/-^ mice (Fig. 5C). As previously shown [10] [11,21], these mice had increased bacterial burdens in the organs (Fig. 5D), another indication that anti-*Ft* IgM is protective.

### Protective IgM is specific for *Ft* LPS

**Fig 6:**
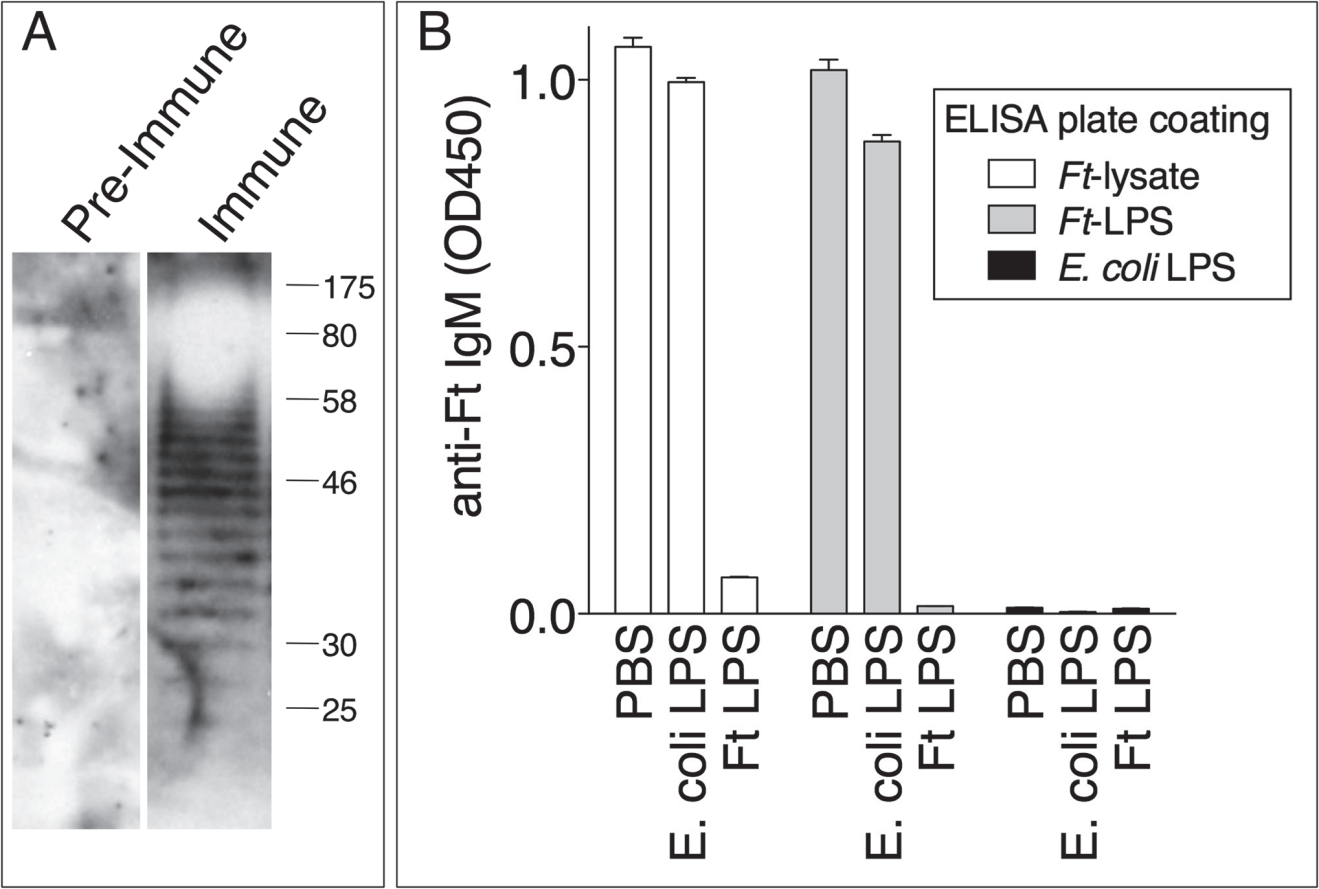
Protective anti-*Ft* IgM are specific for *Ft* LPS. (A) Preimmune serum or day 7 *Ft*-immune serum were used to probe *Ft* lysate by immunoblot. (B) *Ft* immune serum was incubated with PBS, *E*. *coli* LPS, or *Ft* LPS and then used in a competitive ELISA assay on plates coated with *Ft* lysate, *Ft* LPS, or *E*. *coli* LPS. One representative experiment of two is shown. Data are expressed as mean + S.D.

Natural antibodies belonging to the IgM class are present in serum regardless of exposure to pathogens and tend to be poly-reactive and specific for T-independent antigens such as bacterial lipopolysaccharides [37]. As shown in Fig. 6A, pre-immune serum did not contain IgM reactive against a *Ft* LVS lysate. In contrast, the immune serum reacted with the *Ft* LVS lysate in a banding pattern characteristic of LPS. Competitive ELISA was used to show that the anti-*Ft* IgM was specifically directed against *Ft* LPS but did not react with *E*. *coli* LPS (Fig. 6B), reinforcing the notion that this is an *Ft*-specific response and that the measured anti-*Ft* LPS IgM is not part of the natural antibody repertoire.

### Anti-*Ft* IgM efficiently agglutinates *Ft* LVS and promotes phagocytosis

**Fig 7:**
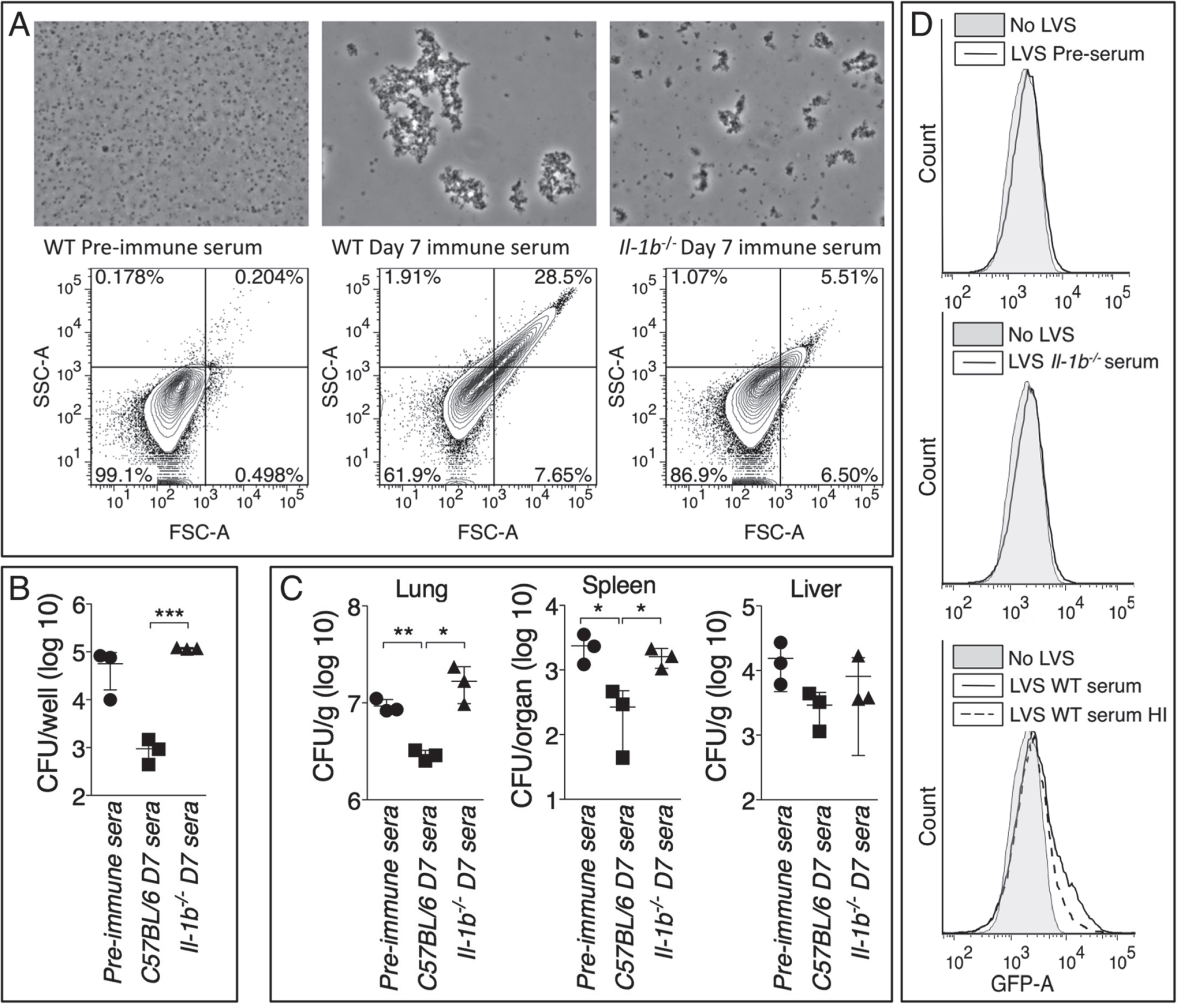
Anti-Ft IgM efficiently agglutinates *Ft* and promotes phagocytosis. (A) *Ft* LVS agglutinated with the indicated sera was examined by microscopy or flow cytometry. (B) Agglutinated *Ft* LVS was used to infect BMM and intracellular bacteria replication was measured 18 hours later. (C) Agglutinated *Ft* LVS (6.5x10^3^ CFU) was used to infect intranasally C57BL/6J mice and bacteria burdens were measured 48 hours later. (D) GFP-expressing *Ft* LVS was agglutinated with the indicated sera and used to infect BMDC. Bacteria uptake was measured by flow cytometry. HI, heat inactivated. Data are expressed as mean + S.D. *p<0.05, **p<0.01, ***p<0.001. Unpaired t-test.

Complement fixation and agglutination are among the most effective antibacterial function of IgM. *Ft* LVS is known to be resistant to complement-mediated lysis [38] [39]. However, IgM-mediated C3 opsonization has been shown to enhance *Ft* LVS phagocytosis by PMN [40]. Day-7 immune serum from *Ft* LVS-infected C57BL/6 mice efficiently agglutinated *Ft* LVS *in vitro*, a phenomenon not observed with pre-immune serum and only partially with serum from infected *Il-1b* ^-/-^ mice (Fig. 7A). Pre-incubation of *Ft* LVS with immune serum significantly reduced infectivity and intracellular replication in bone marrow-derived macrophages (BMM) (Fig. 7B) or in C57BL6/J mice (Fig. 7C). Pre-agglutinated bacteria were also phagocytosed more efficiently by bone marrow-derived dendritic cells (BMDC) (Fig. 7D). When GFP-expressing *Ft* LVS was pre-incubated with pre-immune serum or serum from infected *Il-1b* ^-/-^ mice only 9.8% or 8.75% of BMDC phagocytosed bacteria and became GFP-positive, respectively. In contrast, agglutination of *Ft* LVS with serum from infected C57BL/6J mice dramatically increased phagocytosis of the bacteria (25.3% of cells were GFP-positive). Heat inactivation of this serum reduced the percentage of cells containing bacteria (16.5%) suggesting that the opsonic function of anti-*Ft* IgM is partially mediated by complement activation, a result consistent with the above-mentioned role of C3 in *Ft* LVS uptake. Pre-agglutination did not affect growth of bacteria in liquid cultures (S2 Fig.). Taken together, these results suggest that agglutination and C3-mediated opsonization are the main mechanisms responsible for the protective effect of anti-*Ft* IgM.

### Role of B1a B cells in production of anti-*Ft* LPS IgM

**Fig 8:**
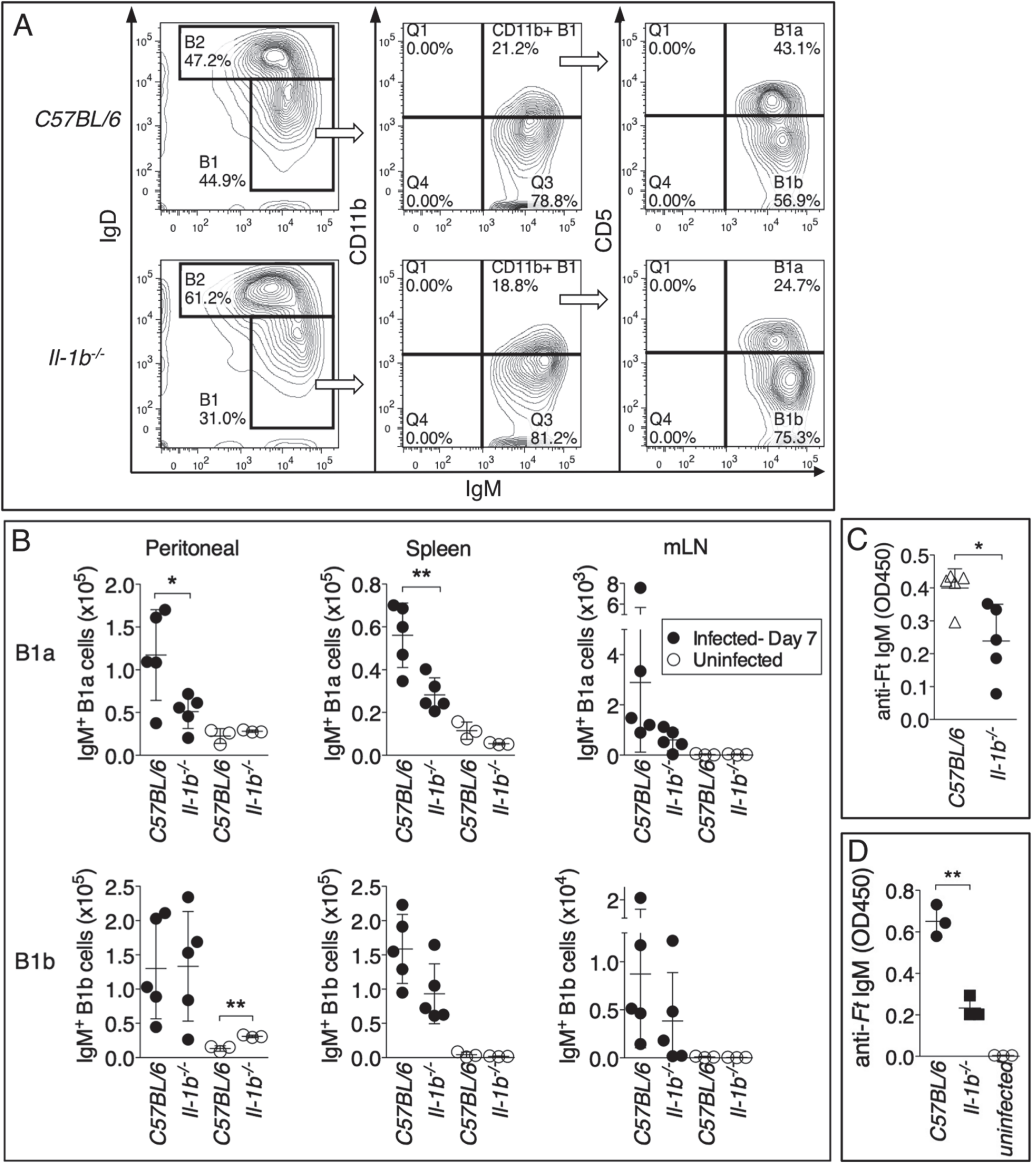
Reduced number of B1a B cells in *Il-1b* ^-/-^ mice infected with *Ft* LVS. (A) Representative FACS profile and gating strategy to identify B1a and B1b B cells. (B) The number of B1a and B1b B cells was measured in peritoneal lavage, spleen, and mediastinal lymph nodes of indicated mouse strains intranasally infected with *Ft* LVS 10^3^ CFU 6 days p.i. (C) Total spleen cells (5x10^6^ cells/ml) or (D) purified B1a B cells (x10^6^ cells/ml) from mice infected as in A were cultured for 24 hours. IgM was measured in culture supernatants. One representative experiment of three is shown. Data are expressed as mean + S.D. *p<0.05, **p<0.01. Unpaired t-test.

Both B1 and B2 B cells subsets are known to contribute to rapid production of IgM during infection and to a different extent depending on the pathogen. B1 B cells and marginal zone B cells (MZ B cells) are a major source of natural antibodies and IgM specific for T-independent antigens such as LPS and self-antigens [41] [42]. Previous work has shown that immunization with purified *Ft* LPS caused expansion of B1a B cells [43]. Analysis of various B cells subsets in mice infected for 7 days with *Ft* LVS revealed that C57BL/6J and *Il-1r1* ^-/-^ mice or *Il-1b* ^-/-^ mice (S3 Fig.) had a similar percentage and total number of B cells, MZ B cells, and follicular B cells in different anatomical locations. In contrast, B1a B cells, but not B1b B cells, were detected in lower proportions in the peritoneal cavity and spleen of *Il-1b* ^-/-^ mice, compared to C57BL/6J mice (Fig. 8B). B1a B cells were present in equal amount in uninfected C57BL/6J or *Il-1b* ^-/-^ mice suggesting that absence of IL-1β affects the infection-induced expansion of these cells, not their homeostasis. Spleen cells from infected C57BL/6J and *Il-1b* ^-/-^ mice were cultured for 18 hours in presence of PMA and anti-*Ft* IgM was measured in the cultured supernatants. As shown in Fig. 8C, cells derived from *Il-1b* ^-/-^ mice released significantly lower amount of anti-*Ft* IgM compared to C57BL/6J cells. Given that B1a cells were the only B cell subset differentially represented in the spleen of C57BL/6J and *Il-1b* ^-/-^ mice (Fig. 8B and S3 Fig.), these data suggests that the anti-*Ft* LPS IgM is produced by B1a B cells. To demonstrate this point conclusively, B1a B cells were purified by flow cytometry from the spleen of C57BL/6J or *Il-1b* ^-/-^ infected mice and cultivated for 24 hours. Anti-*Ft* LPS IgM were present in the conditioned supernatants of purified B1a B cell cultures (Fig. 8D) and, more importantly, in higher amount in C57BL/6J than in *Il-1b* ^-/-^ cells. Together with the *in vivo* results, these data demonstrate that B1a B cells are the source of the *Ft*-specific IgM and that expansion of these cells depends on IL-1β.

## Discussion

A number of studies over the past few years have characterized the innate immune response against *Ft* LVS infection and in particular the protective role played by activation of the AIM2 inflammasome [3,44]. However, by comparison, the role of IL-1β and IL-18 in this infection has been somewhat neglected and remains unclear.

Our study is the first to show that during lung infection with *Ft* LVS, IL-1β and IL-18 are protective, but through different mechanisms. IL-18-deficient mice quickly succumbed to infection with high organ bacterial burdens and low levels of IFNγ, a cytokine that has been shown to be critical for protection from tularemia [30] [31] [32] [33]. Administration of IFNγ increased the survival of *Il-18* ^-/-^ mice, suggesting that the lower resistance to tularemia is due to an inability to produce IFNγ. In contrast, IFNγ administration had no effect on *Il-1r1* ^-/-^ mice survival. Thus, different mechanisms determine the increased susceptibility of *Il-1r1* ^-/-^ and *Il-18* ^-/-^ mice to tularemia. This conclusion was also supported by the observation that *Il-1r1* ^-/-^ or *Il-1b* ^-/-^ mice appear to be able to control the infection, at least in its early stages, but eventually succumbed. This raised the possibility that responses at the boundary of innate and adaptive immunity were defective in absence of IL-1β. In fact, our results show that *Il-1r1* ^-/-^ mice produced *Ft* LPS-specific IgM in a significantly reduced amount compared to C57BL/6J mice. Passive immunization experiments showed that anti-*Ft* IgM were protective, suggesting that the higher susceptibility of IL-1-deficient mice is partly due to reduced production of anti-*Ft*-LPS IgM.

The antibody response to *Ft* infection has been characterized in detail in mice and humans and is mainly directed against LPS and composed of IgM and IgG [45]. Several studies have previously shown that *Ft* LVS infection, or immunization with *Ft* LPS, result in a protective humoral response that can be passively transferred [46] [47] [48] [49]. In all these studies, the passive immunization relied on immune sera obtained several weeks post infection/immunization, implying a role for *Ft*-specific IgG, rather than IgM. One study in fact showed that in order to be protective, serum should be collected at least 15 days p.i. [46]. In that study mice were infected intradermaly as opposed to the intranasal route we used. One novelty of our study is that it shows that during intranasal infection with *Ft* LVS the protective humoral response is deployed more rapidly than previously thought and that IgM is a critical component of this response. The fact that *Ft* LVS has been shown to have a significant extracellular phase in infected mice [50] explains why the humoral response can be so effective against infection with this facultative intracellular bacterium. Importantly, our results show that anti-*Ft* IgM is present in the BALF of infected mice suggesting a role for this Ig isotype at mucosal surfaces. It is likely that infection-induced tissue damage and vascular leakage are responsible for IgM spillage into alveolar spaces.

It is increasingly recognized that IgM plays a protective role during infections, yet the mechanisms of protection have not been consistently defined. IgM efficiently fixes complement and promotes bacteria agglutination and phagocytosis. Although, *Ft* is resistant to complement-mediated lysis [39] [51], it has been demonstrated that IgM-mediated C3 deposition enhances *Ft* LVS phagocytosis by neutrophils [40]. Our results support the conclusion that the protection conferred by passive transfer of anti-*Ft* IgM is mediated by enhanced agglutination/opsonization.

Although it is widely accepted that IgM represents an important first line of defense against infection [36], the pathways leading to production of these antibodies during infection and many aspects of their biology remain unclear. Different B cell subsets, including marginal zone (MZ) B cells and B1 B cells, have been shown to be the main source of the IgM rapidly generated against T-independent antigens during viral or bacterial infections [52]. Studies have indicated that among B1 B cells, the B1a subset is primarily responsible for production of natural IgM while B1b B cells produce pathogen-specific immune IgM [53,54]. However, recent studies have challenged this simplistic classification by showing pathogen-specific antibody production by B1a B cells [43].

Here we show that the reduced serum titer of *Ft* LPS-specific IgM in IL-1-deficient mice correlated with a significant reduction in the percentage of B1a B cells in the spleen and peritoneal cavity in these mice. Purification of B1a B cells from infected mice confirmed that these cells are responsible for production of the protective anti-*Ft* LPS IgM. This conclusion is in agreement with the work of Cole *et al*. [43] that elegantly showed that vaccination of mice with purified *Ft* LPS rapidly elicits expansion of a rare population of B1a B cells as well as production of anti-*Ft* LPS protective antibodies. In a follow-up study [55], the same authors showed that the protection conferred by *Ft* LPS immunization required antibody production and macrophages activation. It should be noted that neither study showed whether the protective anti-*Ft* LPS antibodies were IgM and whether transfer of sera containing them was sufficient for protection, as demonstrated in our experiments. Our study complements that of Cole *et al*. [43] by showing that expansion of B1a B cells and the production of anti-*Ft* LPS antibodies occurs during a natural infection with *Ft* LVS, as opposed to the artificial setting of vaccination. In their study [43], Cole *et al*. concluded that generation of the anti-*Ft* LPS antibodies was independent of innate immunity because this response was observed in TLR4- or TLR2-deficient mice. Interestingly, despite similar titers of anti-*Ft* LPS antibodies in C57BL/6J and *Tlr2* ^-/-^ mice, immunization with *Ft* LPS was not protective in *Tlr2* ^-/-^ mice. Of note, *Ft* LPS does not activate TLR4 or TLR2 [5] [6] [7]. On the other hand, *Ft* LVS has been shown to activate TLR2 [10] [12] and, consistent with this, our results show that production of anti-*Ft* LPS IgM, as well as IL-1β, depends on TLR2 and the inflammasome adaptor ASC. Thus, it appears that generation of anti-*Ft* LPS antibodies can proceed in absence of TLR stimulation in the *Ft* LPS vaccination setting. However, during infection with *Ft* LVS, activation of TLR2 and the inflammasome significantly boosts this response in an IL-1β-dependent fashion. The disparities between vaccination and natural infection are likely due to the fact that vaccination with purified LPS delivers a signal sufficiently strong to activate B1a B cells even in absence of TLR engagement and cytokine production. In contrast, in the natural infection setting “free LPS” may be present in much lower amount and, under these circumstances, the adjuvant effect of IL-1β becomes essential for efficient expansion of B1a B cells. Interestingly, it has been reported that mice deficient in Bruton’s tyrosine kinase, which lack B1a B cells, were more susceptible to lung infection with the *Ft* virulent strain SchuS4 due to an increase in macrophages and NK/NKT cells [56].

The role of IL-1 for antibody production has been extensively analyzed in conventional B cells, and seems to vary according to the experimental setting [57]. In contrast, whether IL-1 regulates the development of B1 B cells and production of IgM by this cell type has not been investigated. Decreased IgM production in *Il-1r1* ^-/-^ mice has been reported, although the involvement of B1 B cells subsets was not examined in that study [58]. Our study reveals that rapid production of anti-*Ft* LPS IgM by B1a B cells depends on IL-1β and suggests, for the first time, a role for IL-1β in the development of B1a B cells during a bacterial infection. Several questions are prompted by our study: Is IL-1β action on B1a B cells direct or mediated by other factors/cells that may regulate migration and activation of these cells? Is the requirement for IL-1β absolute or limited to an enhancing role for a timely production of IgM by B1a B cells? Although the B1a cell population was the only one quantitatively altered in absence of IL-1β, is production of IgM by B1b or MZ B cells regulated by IL-1β in other settings? The IL-1 family member IL-33 has been shown to be required for B1 B cells development [59]. Interestingly, IL-1RAcP, the signaling subunit shared by IL-1 receptor and ST2, the IL-33 receptor, was differentially expressed by B1a and B1b subsets. Whether IL-1RI is differentially expressed in B cell subsets remains to be determined. Future studies should examine the role of IL-1β in B1 B cells activation and IgM production during different bacterial and viral infections

## Materials and Methods

### Ethics statement

All the animal experiments described in the present study were conducted in strict accordance with the recommendations in the *Guide for the Care and Use of Laboratory Animals* of the National Institutes of Health. All animal studies were conducted under protocols approved by the Rosalind Franklin University of Medicine and Science Institutional Animal Care and Use Committee (IACUC) (protocol # B12-07). All efforts were made to minimize suffering and ensure the highest ethical and humane standards.

### Mice

C57BL/6, *Tlr2* ^-/-^, *Il-1r1* ^-/-^, *Il-18* ^-/-^ were purchased from Jackson lab and bred in our facility. *Il-18* ^-/-^-*Il-1r1* ^-/-^ double deficient mice (DKO) were obtained by crossing the parental single knockout mice. *Asc* ^-/-^ mice were obtained from V. Dixit (Genentech). *Il-1a* ^-/-^ and *Il-1a* ^-/-^/*Il-1b* ^-/-^ were from I. Iwakura, *Il-1b* ^-/-^ from D. Chaplin. All mouse strains were on C57BL/6 genetic background and were bred under specific pathogen-free conditions in our facility. Age-(8–12 weeks old) and sex-matched animals were used in all experiments. Generally, experimental groups were composed of at least 5 mice. All the animal experiments described in the present study were conducted in strict accordance with the recommendations in the *Guide for the Care and Use of Laboratory Animals* of the National Institutes of Health. All animal studies were conducted under protocols approved by Institutional Animal Care and Use Committees of the Rosalind Franklin University of Medicine and Science.

### Bacteria, mice infection and treatments

For all experiments the *Francisella tularensis* LVS was used. GFP-expressing *Ft* LVS was provided by Mark Miller (UTHSC). Bacteria were grown in MH broth (Muller Hinton supplemented with 0.1% glucose, 0.1% cysteine, 0.25% ferric pyrophosphate, and 2.5% calf serum) to mid-logarithmic phase, their titer was determined by plating serial dilutions on complete MH agar, and stocks were maintained frozen at −80°C. No loss in viability was observed over prolonged storage. For infections, frozen stocks were diluted in sterile PBS to the desired titer. Aliquots were plated on complete MH agar to confirm actual cfu. Mice were anesthetized with isoflurane using a Surgivet apparatus and 50 ml of bacteria suspension were applied to the nare. In some experiments, mice were injected i.p. daily with recombinant mouse IL-1β (1 μg) or IFNγ (1 μg). IL-1ra (Biovitrum) was administered by alternating s.c. and i.p. injections every 12 hours (60 mg/kg body weight).

### Determination of bacteria growth in tissue culture and organs

Organs aseptically collected were weighted and homogenized in 1 ml PBS containing 0.5% saponin and 3% BSA. Serial dilutions were plated on complete MH agar plates.

### BALF collection and cytokine measurements

BALF were collected from euthanized mice by intratracheal injection and aspiration of 1 ml PBS. Cytokine levels in BALF or serum were measured by ELISA using the following paired antibodies kits: mIL-1α, mIL-1β, mIFNγ (eBioscience), mIL-18 (MBL Nagoya, Japan).

### Determination of antibody titers and competitive ELISA

Blood was collected aseptically from the submandibular vein. *Ft* LPS-specific immunoglobulin levels in sera or BALF were measured by ELISA. Serial dilutions of sera were plated in 96 wells plates coated with *Ft* LVS lysate (10 μg/ml). HRP-conjugated rat anti-mouse IgM or IgG (Southern Biotech Associates, Birmingham, AL) was added followed by TMB substrate and measurement of absorbance at 450 nm. For competitive ELISA (Fig. 6B) plates were coated with *Ft* LVS lysate (10 μg/ml), purified *Ft* LVS LPS (10 μg/ml), or *E*. *coli* LPS (0111:B4, 10 μg/ml). Immune serum was preincubated with PBS, *E*. *coli* LPS or *Ft* LVS LPS (1 μg) for 30 minutes before dilution and addition to coated plates.

### Western blot

*Ft* LVS lysate (50 μg) was separated by 12% PAGE electrophoresis, transferred to PVDF membranes, and probed with pre-immune or immune serum (1:300 dilution) followed by anti-mouse IgM-HRP (Upstate Biotechnologies) and developed by ECL Femto (Pierce).

### Passive immunization

Preimmune serum or sera from different mouse strains infected for 7 days were dialyzed against PBS (100 kDa mw cut-off) and 300 μl were intraperitoneally administered to mice 18 hours before infection. To deplete IgM, serum was incubated 1 hours at 4°C with anti-mouse IgM agarose (Sigma).

### Bacteria agglutination, infectivity, and phagocytosis

*Ft* LVS was grown O/N in complete MH broth. 10 μl of bacterial culture were incubated with 10 μl of serum for 10 minutes at RT. Agglutination was confirmed by microscopy and flow cytometry. To measure infectivity of agglutinated *Ft* LVS *in vitro*, BMM were infected with bacteria preincubated with different sera at a MOI 500 and infection allowed to proceed for 2 hours. Cells were then washed 3 times with PBS and cultured for additional 18 hours in DMEM-10% FCS containing gentamicin and kanamycin (400 μg/ml). Media were aspirated and cells lysed in PBS containing 0.5% saponin and 3% BSA. Serial dilutions were plated on complete MH agar. To measure phagocytosis, BMDC were incubated for 4 hours with GFP-expressing *Ft* LVS agglutinated with different sera. Some sera were heat inactivated at 56 C for 30 minutes. Gentamicin and kanamycin (400 μg/ml) were then added to the medium for 4 more hours to kill extracellular bacteria and cells were analyzed by flow cytometry. The percentage of GFP positive cells (those that phagocytosed *Ft* LVS) was calculated by Overton subtraction.

### Flow cytometry

Cells obtained from BALF, peritoneal lavage, spleen, or mediastinal lymph nodes were resuspended in FACS buffer (1% BSA, 0.05% NaN3 in PBS) containing anti-CD16/CD32 (clone 2.4G2) and counted. For analysis cells were incubated for 30’ on ice with the following antibody cocktails: for myeloid cells, CD11b-PECy7, Ly6G-PE, F4/80-APC, CD11c-FITC and NKp46-BV421; for GC B cells, B220-PercpCy5.5, CD4-APC, FAS-PE and GL-7-FITC; for marginal zone (MZ) and Follicular B cells, CD19-BV421, CD23-PECy7 and CD21-APC; for B1 cells, CD19-BV421, IgM-PECy7, IgD-APC, CD11b-AF700 and CD5-PE; for T_FH_ cells, CXCR5-biotin followed by streptavidin-APC, CD4-PercpCy5.5, BTLA-PE and PD1-FITC.

GC B cells are defined as CD4^-^ B220^+^ cells expressing both FAS and GL-7; T_FH_ cells are CD4^+^ CXCR5^+^ BTLA^+^ [60]; Fol B cells are defined as CD19^+^ CD23^+^ CD21^-/intermediate^; MZ B cells are the CD19^+^ CD21^+^ CD23^-/low^ cells [61]. B cells (CD19^+^) were gated on lymphocyte gate followed by singlet gate (FSC-A vs FSC/H) and identified as B2 or B1 cells according to the surface expression of IgD or IgM, respectively. B1 B cells subsets are defined as described in [54] [61] as CD19^+^ IgM^high^ IgD^-/low^. B1 B cells were selected and gated for the expression of CD11b and CD5. B1a cells are defined as CD5^+^, B1b cells as CD5^-^. FMO or isotype controls were used to set the gate. Data were acquired with a BD LSR II flow-cytometer (BD biosciences) and analyzed with FlowJo 7.6.5 software (Treestar Inc).

### B1a B cell sorting and purification

B1a B cells were purified from the spleen of mice infected for 7 days with *Ft* LVS according to [56]. Single cell suspensions of splenic cells were enriched by negative selection for B cells using the B cell isolation kit (StemCell). The CD19^+^/CD5^+^ B1 a B cells were sorted with a BD FACSAria II u flow cytometer. B1a B cells were resuspended in DMEM-10% FCS at a density of 1.5 x10^6^ and cultured in 96 well plate for 24 hours.

### Statistical analysis

All data were expressed as mean + S.D. Survival curves were compared using the log rank Kaplan-Meier test. Mann-Whitney U test or unpaired t-test were used for analysis of the rest of data as specified in figure legends. Significance was set at *p*<0.05. Statistical analyses were performed using the GraphPad Prism 5.0.

## Supporting Information

S1 Fig Anti-*Ft* IgG and IgA. *Ft*-specific IgG in serum or IgA in BALF of mice intranasally infected with *Ft* LVS 10^3^ CFU were measured on day 7 p.i.
(JPG)

S2 Fig Agglutination of *Ft* LVS does not affect in vitro bacterial growth. *Ft* LVS agglutinated with the indicated sera were grown in complete MH broth and absorbance was measured at indicated time points.
(JPG)

S3 Fig Myeloid and lymphoid cell populations in C57BL/6 and *Il-1r1* ^-/-^ mice. Leukocyte populations were measured in BALF, spleen, and mediastinal lymph nodes of *Il-1r1* ^-/-^ (A-C) or *Il-1b* ^-/-^ (D, E) mice intranasally infected with *Ft* LVS 10^3^ CFU 6 days p.i. One representative experiment of three is shown. Data are expressed as mean + S.D. *p<0.05, **p<0.01. Unpaired t-test.
(JPG)
