# Supplementary figures and images for: Production of Anti-LPS IgM by B1a B Cells Depends on IL-1β and Is Protective against Lung Infection with Francisella tularensis LVS
Source: PLoS Pathog. 2015 Mar 13;11(3):e1004706. doi: 10.1371/journal.ppat.1004706 (PMC4358995; doi:10.1371/journal.ppat.1004706)

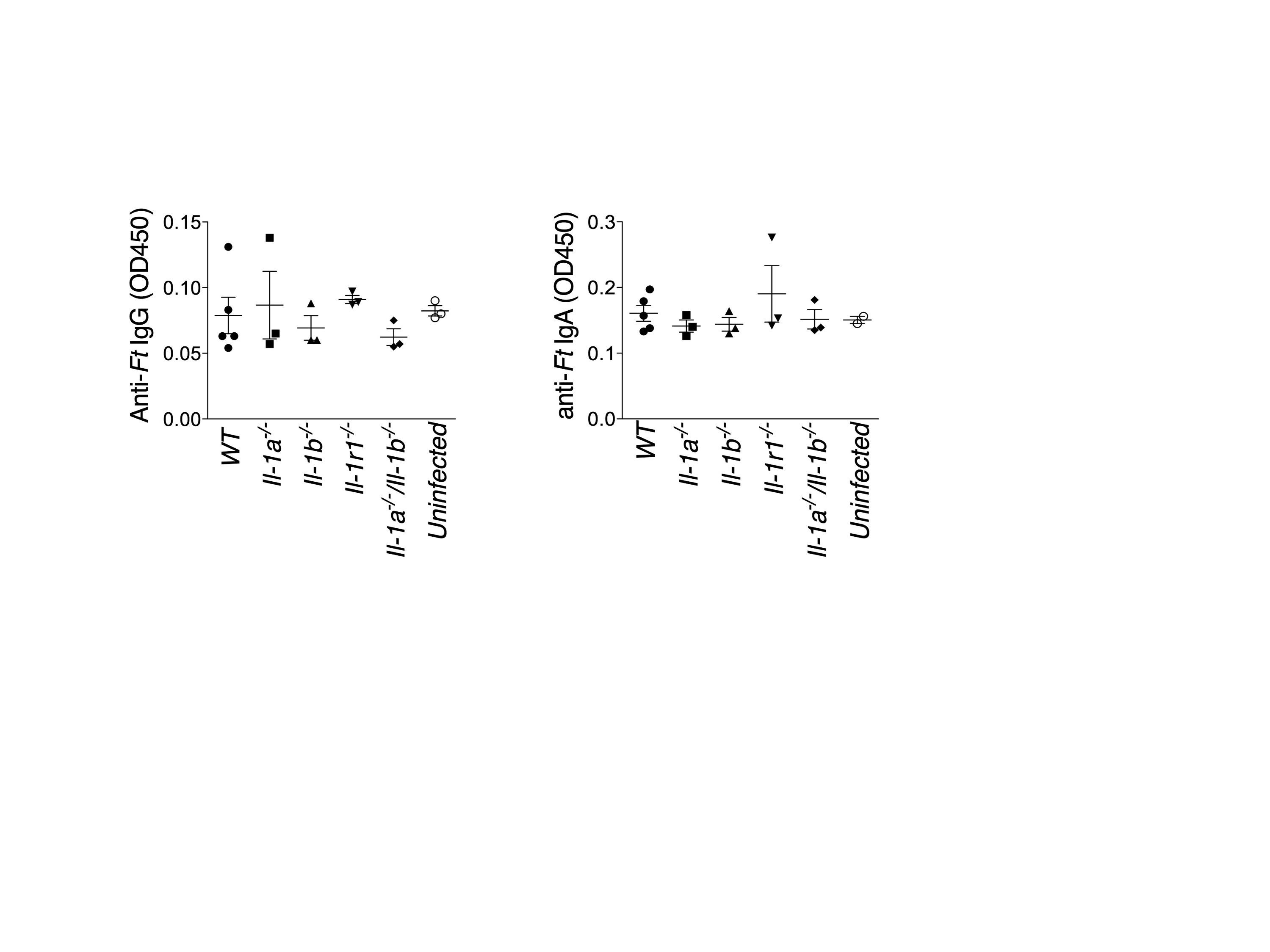

Supplement: S1 Fig — Ft-specific IgG in serum or IgA in BALF of mice intranasally infected with Ft LVS 103 CFU were measured on day 7 p.i. (JPG) [file ppat.1004706.s001.jpg]

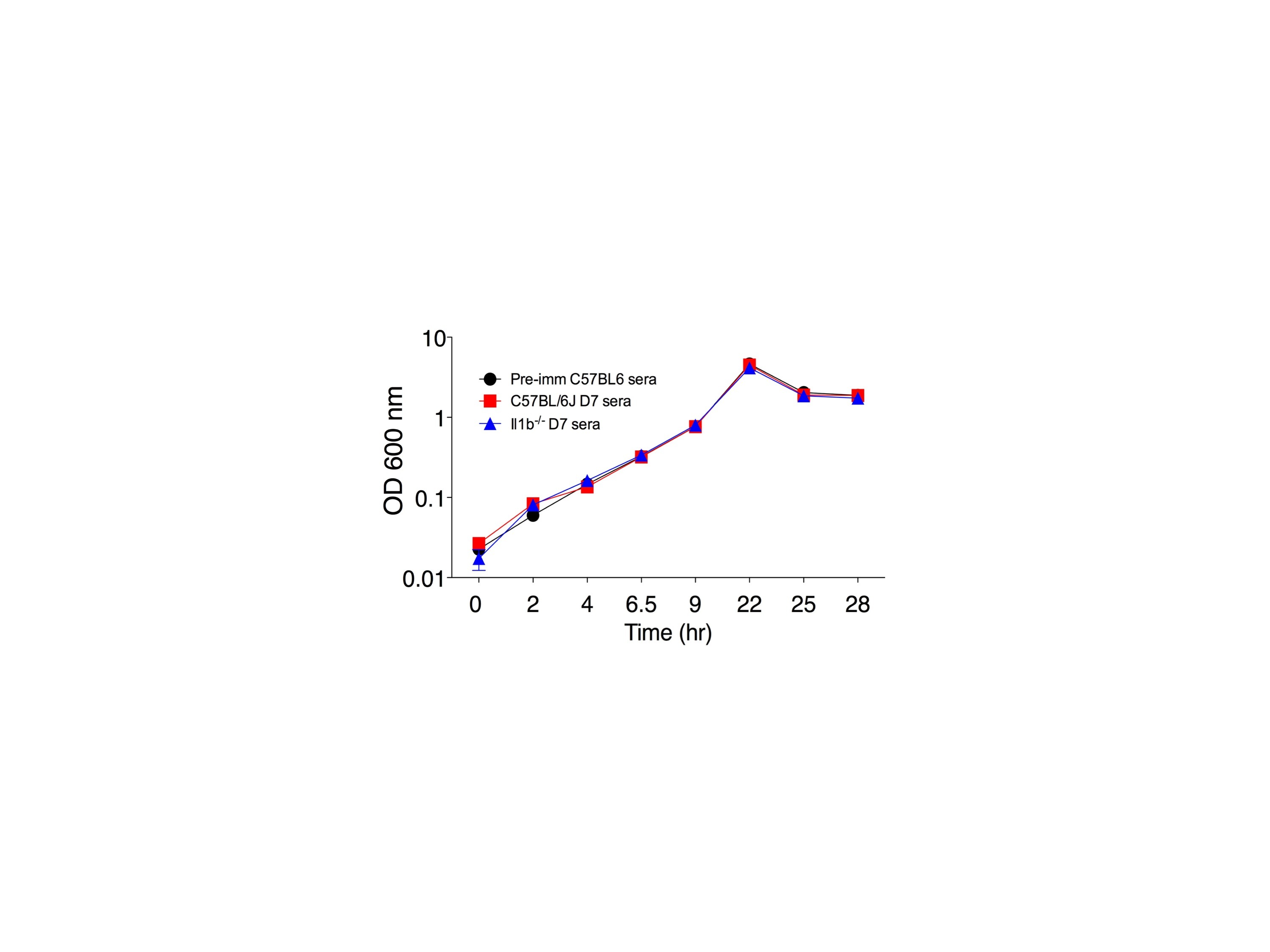

Supplement: S2 Fig — Ft LVS agglutinated with the indicated sera were grown in complete MH broth and absorbance was measured at indicated time points. (JPG) [file ppat.1004706.s002.jpg]

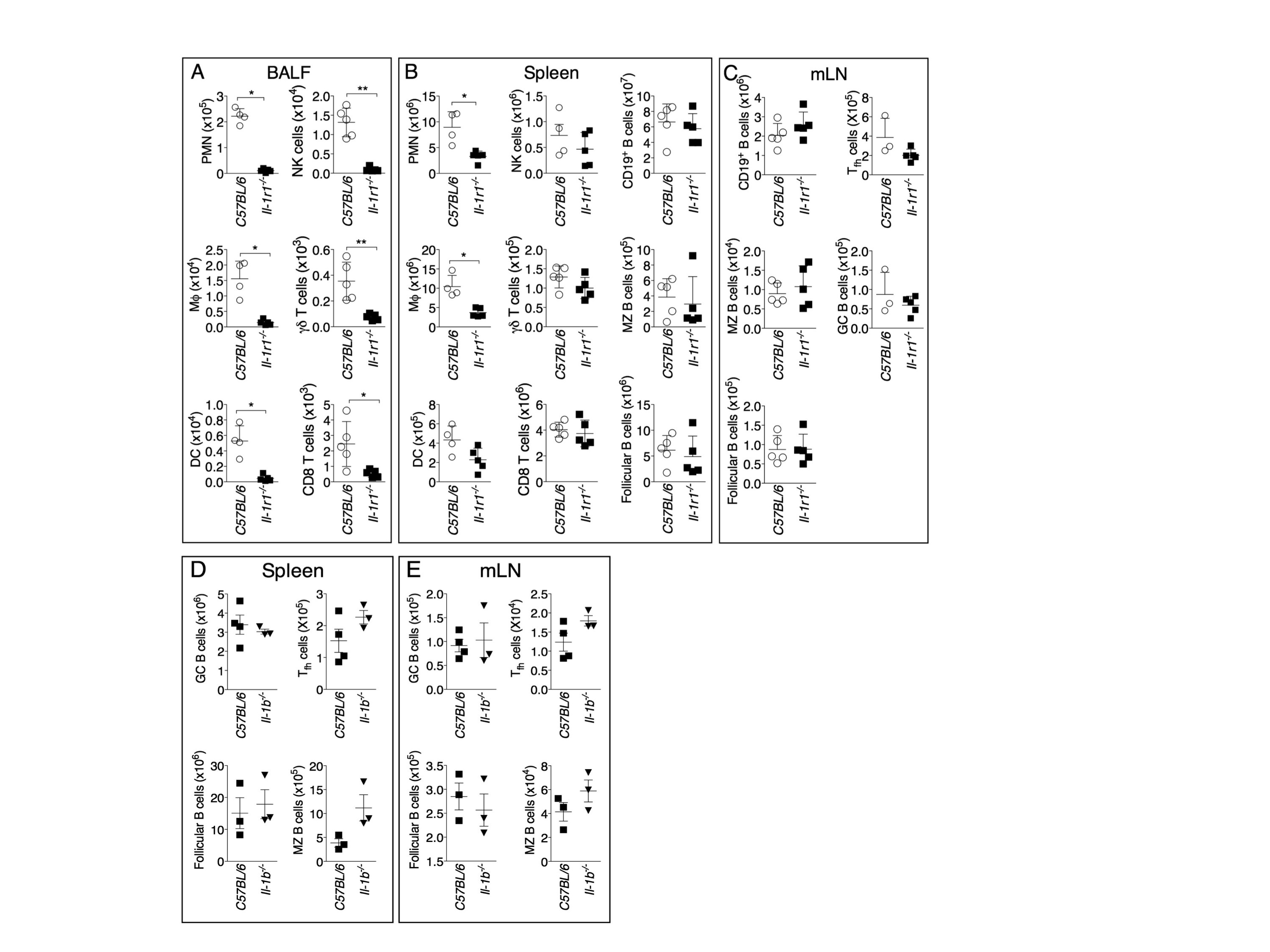

Supplement: S3 Fig — Leukocyte populations were measured in BALF, spleen, and mediastinal lymph nodes of Il-1r1 -/- (A-C) or Il-1b -/- (D, E) mice intranasally infected with Ft LVS 103 CFU 6 days p.i. One representative experiment of three is shown. Data are expressed as mean + S.D. *p<0.05, **p<0.01. Unpaired t-test. (JPG) [file ppat.1004706.s003.jpg]
